# Evaluation of the Expression of Amine Oxidase Proteins in Breast Cancer

**DOI:** 10.3390/ijms18122775

**Published:** 2017-12-20

**Authors:** Woo Young Sun, Junjeong Choi, Yoon Jin Cha, Ja Seung Koo

**Affiliations:** 1 Department of Surgery, Daejeon St. Mary’s Hospital, College of Medicine, The Cathololic University of Korea, Seoul 06591, Korea; sun2729@naver.com; 2 College of Pharmacy, Yonsei Institute of Pharmaceutical Sciences, Yonsei University, Incheon 21988, Korea; junjeong@gmail.com; 3 Department of Pathology, Yonsei University College of Medicine, Seoul 03722, Korea; yooncha@yuhs.ac

**Keywords:** amine oxidase, breast cancer, estrogen receptor, human epidermal growth factor receptor 2, progesterone receptor

## Abstract

We aimed to evaluate the expression of amine oxidase proteins in breast cancer and their clinical implications. We performed immunohistochemical staining of amine oxidase proteins (LOX, lysyl oxidase, AOC3, amine oxidase, MAOA, monoamine oxidase A, MAOB, monoamine oxidase B). Based on their hormone receptors, such as estrogen receptor (ER) and progesterone receptor (PR), human epidermal growth factor receptor 2 (HER-2), and Ki-67 immunohistochemical staining, breast cancer was divided into four molecular subtypes: luminal A, luminal B, HER-2 type, and triple-negative breast cancer (TNBC). Luminal A was observed in 380 cases (49.4%), luminal B in 224 (29.1%), HER-2 type in 68 (8.8%), and TNBC in 98 (12.7%). Stromal AOC3, MAO-A, and MAO-B expression varied according to molecular subtypes. Stromal AOC3 expression was high in luminal B and HER-2 type and MAO-A expression was high in luminal A and luminal B (*p* < 0.001). MAO-B expression was higher in TNBC than in other subtypes (*p* = 0.020). LOX positivity was associated with high histological grade (*p* < 0.001) and high Ki-67 labeling index (LI) (*p* = 0.009), and stromal AOC3 positivity was associated with high histological grade (*p* = 0.001), high Ki-67 LI (*p* < 0.001), and HER-2 positivity (*p* = 0.002). MAO-A positivity was related to low histological grade (*p* < 0.001), ER positivity, PR positivity (*p* < 0.001), and low Ki-67 LI (*p* < 0.001). In univariate analysis, MAO-A positivity was related to short disease-free survival in HER-2 type (*p* = 0.013), AOC3 negativity was related to short disease-free survival and overall survival in ER-positive breast cancer, PR-positive breast cancer, HER-2-negative breast cancer, and lymph node metastasis. In conclusion, the expression of amine oxidase proteins varies depending on the molecular subtype of breast cancer. Stromal AOC3 expression was high in luminal B and HER-2 type, and MAO-A expression was high in luminal A and luminal B.

## 1. Introduction

Amine oxidase is an enzyme that catalyzes the oxidative cleavage of alkylamine into aldehydes and ammonia. Amine oxidase is divided into two groups according to cofactor: lysyl oxidase (LOX) and primary-amine oxidase, copper containing 2 and 3 (AOC2 and AOC3) that are proteins with copper as a cofactor, and monoamine oxidases (MAO), consisting of enzymes MAO-A and MAO-B, with falvin as a cofactor [1]. Amine oxidase participates in various metabolic pathways and is involved in cell differentiation, cell growth, wound healing, detoxification, and cell signaling [2]. Some amine oxidases have been reported to be involved in regulating cancers; LOX has played an important role in colorectal cancer cell dissemination in the bone marrow [3], and expression of AOC3 is associated with cancer prognosis in various tumors [4,5,6]. Altered MAO-A expression is associated with the poor prognosis of cancer and demonstrates a correlation with prostate tumorigenesis and cancer metastasis [7], and MAO-B is known to exhibit high expression in gliomas [8]. Therefore, amine oxidase can be expected to affect cancer biology.

Breast cancer is a representative heterogeneous tumor, with various clinical, histological, molecular, and genetic signatures, and research to classify breast cancer with similar properties is ongoing. Breast cancer has been divided into normal, basal-like, luminal A, luminal B, and human epidermal growth factor receptor 2 (HER-2) type by gene profiling analysis [9,10,11]. In addition to their classification by gene profiling analysis, was divided breast cancer according to estrogen receptor (ER), progesterone receptor (PR), and HER-2 into another subgroup, triple-negative breast cancer (TNBC). TNBC is defined when immunohistochemistry for ER, PR, HER-2, and FISH for HER-2 is negative [12]. These molecular aspects can differ even in metabolic features because of the differences in histological findings, clinical findings, therapeutic response, and prognosis. Basal-like type and TNBC are reported to exhibit high expression of GLUT-1 and CAIX [13,14], and glutaminolysis-related proteins are reported to be highly expressed in HER-2 type breast cancer [15]. These findings support the relationship between metabolism and molecular subtype. However, research on the activity of amine oxidase proteins in breast cancer has not yet been conducted. This study aimed to investigate the expression and clinical implication of amine oxidase proteins in human breast cancer.

## 2. Results

### 2.1. Patient Characteristics

Among 770 cases, luminal A was observed in 380 cases (49.4%), luminal B in 224 cases (29.1%), HER-2 in 68 cases (8.8%), and TNBC in 98 cases (12.7%). There was a difference in age (*p* = 0.040), histological grade (*p* < 0.001), nodal metastasis status (*p* = 0.028), and Ki-67 labeling index (LI) (*p* < 0.001) with respect to the molecular subtype. High histological grade and Ki-67 LI were associated with TNBC, high percentage of old-aged patients was associated with HER-2, and increased nodal metastasis was associated with luminal A (Table 1).

### 2.2. Differential Expression of Amine Oxidase in Different Tumor Subtypes

**Figure 1:**
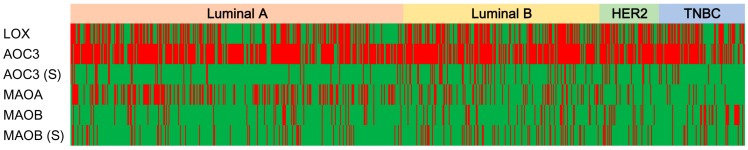
Heat map of amine oxidase in breast cancer molecular subtype. LOX, lysyl oxidase, AOC3, amine oxidase, MAOA, monoamine oxidase A, MAOB, monoamine oxidase B, TNBC, triple negative breast cancer, S, stroma, green, positive, red, negative.

**Figure 2:**
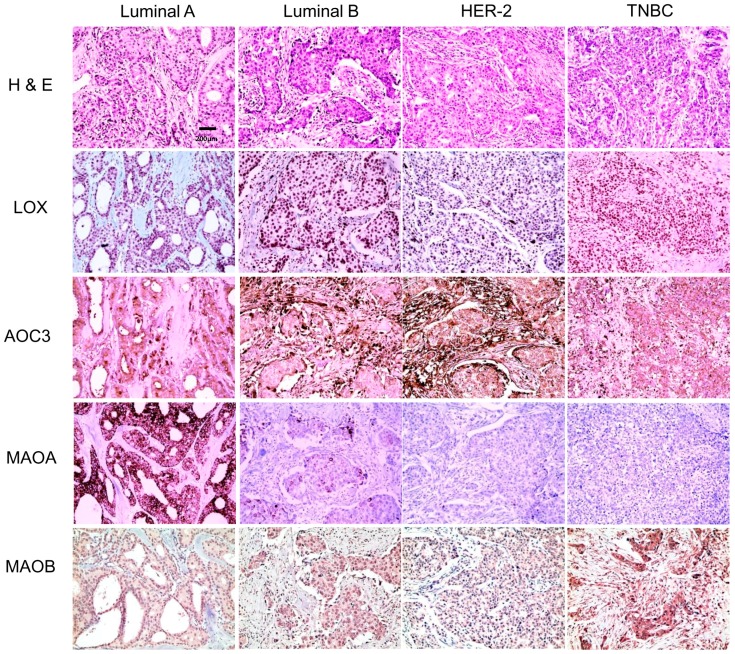
Differential expression of amine oxidase in different tumor subtypes. The expression of stromal AOC3, MAO-A, and MAO-B; high expression of stromal AOC3 in luminal B and HER-2-type breast cancers; and high MAO-A expression in luminal A and luminal B (*p* < 0.001). MAO-B expression was higher in TNBC than that in other proteins (*p* = 0.020).

Investigation of the expression of the amine oxidase family of proteins according to the molecular subtype of breast cancer revealed differences in the expression of stromal AOC3, MAO-A, and MAO-B; high expression of stromal AOC3 in luminal B and HER-2-type breast cancers; and high MAO-A expression in luminal A and luminal B (*p* < 0.001). MAO-B expression was higher in TNBC than that in other proteins (*p* = 0.020) (Table 2, and Figure 1 and Figure 2).

### 2.3. Correlation of the Expression of Amine Oxidase Proteins in Breast Cancer

A statistically significant correlation was observed among amine oxidase proteins: LOX–AOC3 (*r* = 0.238, *p* < 0.001), LOX–AOC3 (S) (*r* = 0.144, *p* < 0.001), LOX–MAO-B (S) (*r* = 0.160, *p* < 0.001), AOC3 (S)–MAO-B (S) (*r* = 0.121, *p* < 0.001), AOC3–AOC3 (S) (*r* = 0.160, *p* < 0.001), and MAO-B–MAO-B (S) (*r* = 0.245, *p* < 0.001) (Table 3).

### 2.4. Correlation between the Expression of Amine Oxidase and Clinicopathological Characteristics

**Figure 3:**
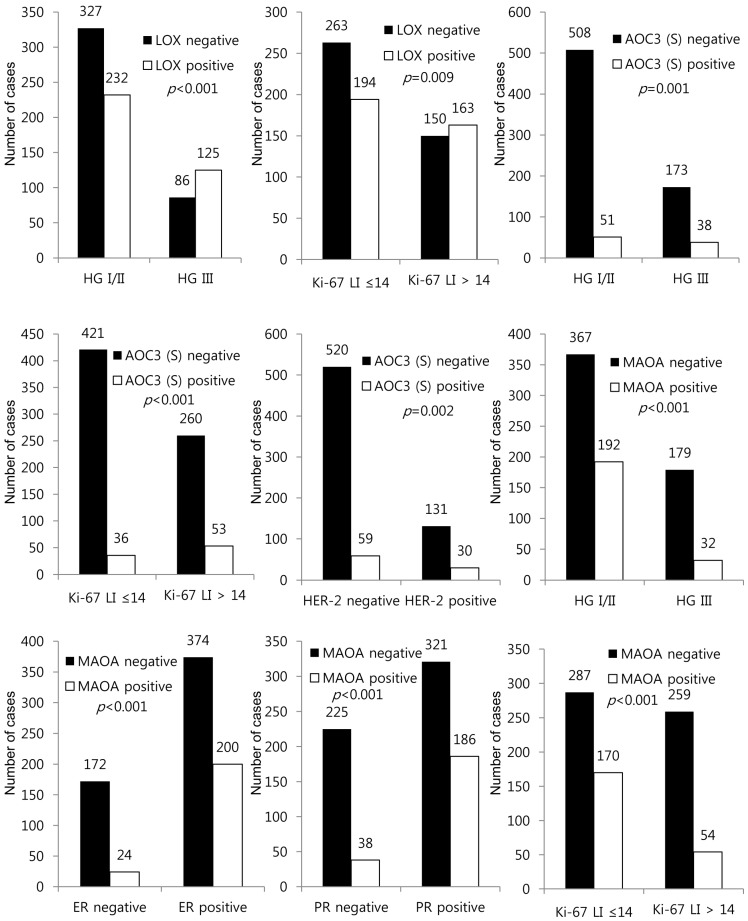
Correlation between the expression of amine oxidase and clinicopathological characteristics. LOX positivity was associated with a high histological grade (*p* < 0.001) and high Ki-67 LI (*p* = 0.009). Stromal AOC3 positivity was associated with a high histological grade (*p* = 0.001), high Ki-67 LI (*p* < 0.001), and HER-2 positivity (*p* = 0.002). MAO-A positivity was associated with a low histological grade (*p* < 0.001), estrogen receptor (ER) positivity (*p* < 0.001), PR positivity (*p* < 0.001), and low Ki-67 LI (*p* < 0.001).

LOX positivity was associated with a high histological grade (*p* < 0.001) and high Ki-67 LI (*p* = 0.009). Stromal AOC3 positivity was associated with a high histological grade (*p* = 0.001), high Ki-67 LI (*p* < 0.001), and HER-2 positivity (*p* = 0.002). MAO-A positivity was associated with a low histological grade (*p* < 0.001), ER positivity (*p* < 0.001), PR positivity (*p* < 0.001), and low Ki-67 LI (*p* < 0.001) (Figure 3).

### 2.5. Functional Analysis Using STRING Database

**Figure 4:**
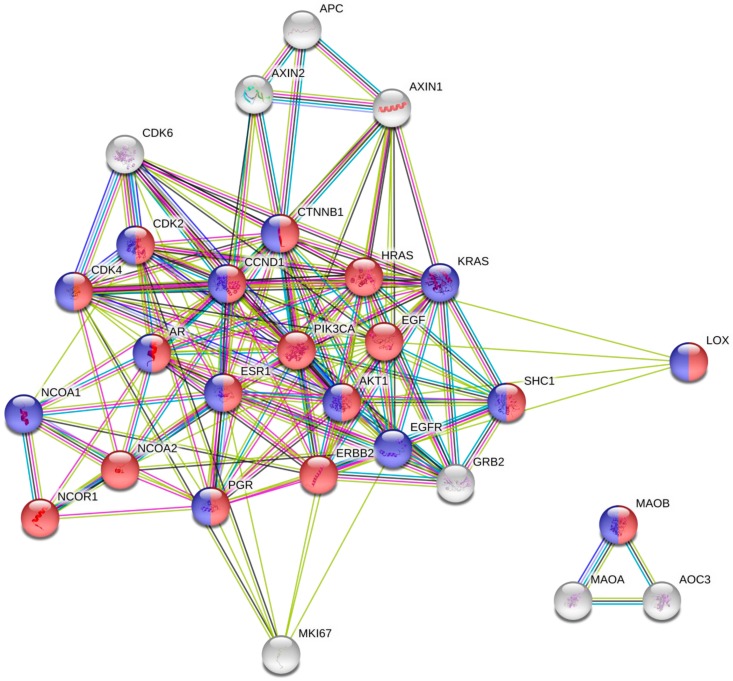
Functional analysis using STRING database. Using “Mutual Exclusivity” function, 3 significant gene pairs with mutually exclusive alteration were identified (*ERBB2-PGR*, *AOC3-ERBB2*, *MKI67-ESR1*) and 1 gene pair with concurrent alteration (*AOC3-MAOA*). The response to hormone stimulus (red) and the response to lipids (blue) include Lox and MAOB together with ESR1, suggestive of the presence of common activator of estrogen positive type of breast cancer.

Using “Mutual Exclusivity” function, 3 significant gene pairs with mutually exclusive alteration were identified (*ERBB2-PGR*, *AOC3-ERBB2*, *MKI67-ESR1*) and 1 gene pair with concurrent alteration (*AOC3-MAOA*) in this set. The response to hormone stimulus and the response to lipids include Lox and MAOB together with *ESR1*, suggestive of the presence of common activator of estrogen positive type of breast cancer (Figure 4). As the tumor fraction of TCGA was at least 60%, the expression assessed may be originated from the tumor, not from the stroma. In addition, we used the “Enrichments” function of cBioPortal and acquired other gene sets that are significantly increased in samples with alteration of studied markers. With this gene set, we performed pathway analysis using PANTHER database. Interestingly, transforming growth factor beta-activated receptor activity (GO: 0005024) and transforming growth factor beta binding (GO: 0050431), and regulation of vascular endothelial growth factor receptor signaling pathway (GO: 0030947) were over-represented in this gene set, suggestive of potential involvement of studied pathway in the epithelial-mesenchymal transition and angiogenesis of breast cancer (Supplementary data).

### 2.6. Effect of the Expression of Amine Oxidase on Survival

**Figure 5:**
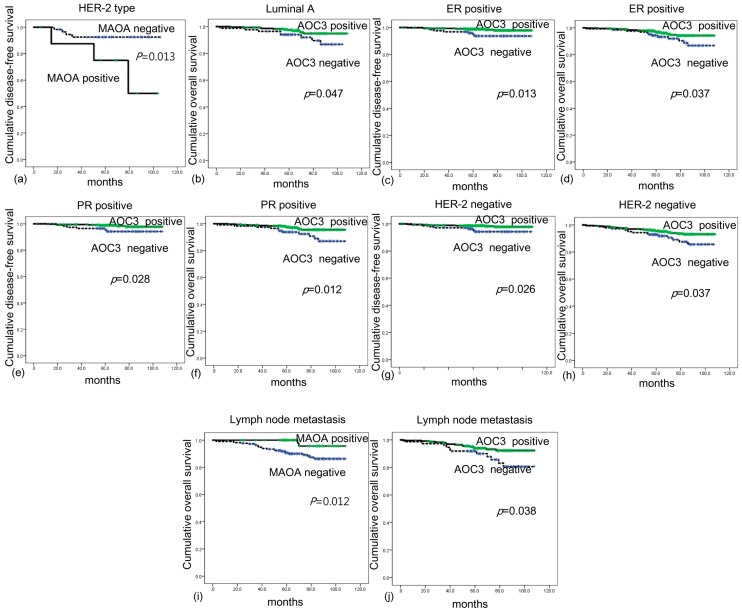
Effect of the expression of amine oxidase on survival in breast cancer. MAO-A positivity was associated with short DFS in HER-2-type breast cancer (**a**) (*p* = 0.013), luminal A was associated with short OS (**b**) (*p* = 0.047). In ER-positive breast cancer, AOC3 negativity was associated with short DFS (**c**) and short OS (**d**) (*p* = 0.013 and *p* = 0.037, respectively). AOC3 negativity was associated with short DFS (**e**) and short OS (**f**) in PR-positive cancer (*p* = 0.028 and *p* = 0.012, respectively). In HER-2-negative breast cancer, AOC3 negativity was associated with short DFS (**g**) and short OS (**h**) (*p* = 0.026 and *p* = 0.037, respectively), and in the breast cancer showing lymph node metastasis, AOC3 negativity was associated with short DFS (**i**) and short OS (**j**) (*p* = 0.038 and *p* = 0.012, respectively).

The analysis of the effect of amine oxidase protein expression on breast cancer prognosis was not significant in the univariate analysis (Table 4). However, in the subgroup analysis, MAO-A positivity was associated with short DFS in HER-2-type breast cancer (*p* = 0.013), luminal A was associated with short OS (*p* = 0.047). In ER-positive breast cancer, AOC3 negativity was associated with short DFS and OS (*p* = 0.013 and *p* = 0.037, respectively). AOC3 negativity was associated with short DFS and OS in PR-positive cancer (*p* = 0.028 and *p* = 0.012, respectively). In HER-2-negative breast cancer, AOC3 negativity was associated with short DFS and OS (*p* = 0.026 and *p* = 0.037, respectively), and in the breast cancer showing lymph node metastasis, AOC3 negativity was associated with short DFS and OS (*p* = 0.038 and *p* = 0.012, respectively) (Figure 5).

## 3. Discussion

We investigated the expression of amine oxidase proteins based on molecular subtypes of breast cancer. We observed that stromal AOC3 was highly expressed in luminal B and HER-2-type breast cancer, and MAO-A was highly expressed in luminal A and luminal B. To the best of our knowledge, ours is the first study to present the results of AOC3 expression in breast cancer. No previous study has examined the expression of AOC3 in breast cancer tissues, and the expression of AOC3 in breast cancer stroma has not yet been reported. AOC3 is considered to be a marker distinguishing myofibroblasts from activated fibroblasts, as reported in a previous study [16]. The subgroup exhibiting a myofibroblastic phenotype in a cancer-associated fibroblast (CAF) has been reported to cause tumor cell motility [17] and collagen fiber elongation [18]. In addition, the stromal cell expressing AOC3 influences tumor biology. There is a difference in the expression of CAF proteins depending on the molecular subtype of breast cancer. In particular, most CAF proteins are reported to be highly expressed in HER-2-type breast cancer stroma, which is consistent with our study [19]. CAF gene expression differs with respect to the molecular subtype of breast cancer [20]. The expression of the gene involved in cancer cell migration was higher in the CAF of HER-2-type breast cancer than that in TNBC and ER-positive type cancer [20]. AOC3 is a marker to determine the myofibroblast phenotype of CAF, and the myofibroblast promotes cancer cell migration and invasion [21]. Therefore, high expression of AOC3 can be associated with high expression of the gene involved in cancer cell migration in HER-2-positive breast cancer stroma.

MAO-A was highly expressed in luminal-type cancer, and the expression of MAO-A was reported to be increased with chemically-induced mammary cancer in rats, in previous studies [22,23]; however, the expression of MAO-A using human breast cancer tissue has not yet been reported. Since all three ER receptors (α, β, and γ) increased the transcription of MAO-A [24], the high expression of MAO-A with ER-positive luminal-type breast cancer is explained in our study. In a study analyzing gene chip data from human cancer tissue, it was reported that MAO-A expression decreased in 95.4% of human cancers compared to that in normal tissue; research on human breast cancer and human basal-like breast cancer demonstrated decreased expression of MAO-A compared with that in normal tissue [25]. However, it is difficult to compare the present study with the previous one, since our study does not analyze the comparison with normal tissue but demonstrates the difference in breast cancer by molecular subtype.

MAO-B was highly expressed in human TNBC in our study. However, there have been no studies evaluating MAO-B expression in human TNBC tissue. Previous studies using breast cancer cell lines have reported that estrogen-related receptor (ERR) increases MAO-B expression, while ER decreases ERR-induced MAO-B expression [26]. Therefore, high expression of MAO-B in TNBC, which is negative for ER, can be predicted. Overexpressing ERRα in MDA-MB-231, which is a TNBC breast cancer cell line, increases MAO-B expression, which supports the results of our study [27].

In the present study, LOX positivity was associated with high histological grade and high Ki-67 LI. A previous study reported that LOX mRNA level was higher in breast cancer tissue than normal tissue; however, there was no difference in LOX mRNA level between ER-positive breast cancer and TNBC, which is consistent with our findings [28]. A previous study reported that LOX is highly expressed in highly invasive cancers and metastatic breast cancer cell lines [29,30]. In addition, LOX expression increases in breast cancer cells under hypoxic conditions [28]. It is well known that breast cancers with a high Ki-67 LI have a high proliferative potential and are likely to be in hypoxic conditions. Therefore, the expression of LOX can be expected to be high in aggressive breast cancers with a high histological grade and high Ki-67 LI.

In this study, AOC negativity with ER-positive breast cancer, PR-positive breast cancer, HER-2-negative breast cancer, and breast cancer with lymph node metastasis demonstrated an association with short DFS and short OS. Studies analyzing breast cancer tissue and benign tissue with proteomic analysis showed that AOC3 expression was decreased in breast cancer [31], suggesting the possibility that the stage-specific marker, AOC3, may affect the prognosis of breast cancer. However, AOC3 overexpression is associated with poor prognosis of astrocytoma, which is different from the results in this study [4]. AOC3 is an endothelial adhesion protein, and is involved in cancer cell extravasation of cells that invade the endothelial cell layer of other organs and tissues, and AOC3 is reported to be associated with poor prognosis [32,33]. However, in previous studies on colon and gastric cancer, serum AOC3 level was higher in cancer patients than in healthy subjects, and a low preoperative level of serum AOC3 indicated poor prognosis [5,6]. This finding supports the results of our study that AOC negativity indicated poor prognosis.

Although it is not possible to evaluate the exact reason for the association of low serum AOC3 level with poor prognosis, tumor immunity can be presented as a possible mechanism. Serum AOC3 promotes lymphocyte binding in endothelial cells and can lead to lymphocyte accumulation in tumor vesicles [34]. This in turn, activates tumor-infiltrating lymphocytes (TIL) to induce a protective local immune response. Therefore, tumor proliferation can be suppressed. In the absence of AOC3 expression in breast cancer, we can assume possibilities of a poor prognosis by low protective, local immune responses. However, further studies investigating this association are needed.

The clinical significance of this study is that amine oxidase proteins can be used for possible targeted therapeutic treatments in breast cancer. In various cancers, the LOX inhibitor [35,36,37,38] and MAO-A inhibitors [39,40,41] have been studied for tumor suppression, and these studies are crucial in breast cancer as well.

In conclusion, the expression of amine oxidase proteins varied depending on the molecular subtype of breast cancer. Stromal AOC3 expression was high in luminal B and HER-2-type breast cancer, and MAO-A expression was high in luminal A and luminal B. Expression of amine oxidase proteins, especially AOC3, is associated with prognosis in breast cancer subgroups.

## 4. Materials and Methods

### 4.1. Patient Selection and Histological Evaluation

We analyzed 770 cases diagnosed as invasive ductal carcinoma (IDC), not otherwise specified (NOS), at Severance Hospital between January 2006 and December 2008. Patients who underwent neoadjuvant chemotherapy or hormonal therapy were excluded from the study.

This study was approved by the Institutional Review Board of Yonsei University Severance Hospital (Approval number: 4-2016-0832, Approved in 14 November, 2016). IRB exempted the informed consent from patients. The sections were stained with hematoxylin and eosin (H&E) and all cases were retrospectively reviewed by one breast pathologist (Koo JS). Histological grade was assessed using the Nottingham grading system [42]. Clinicopathologic parameters evaluated in each case included patient age at initial diagnosis, lymph node metastasis, tumor recurrence, and patient survival.

### 4.2. Tissue Microarray

A representative area showing tumor and tumor stroma was selected on an H&E-stained slide, and a corresponding spot was marked on the surface of the paraffin block. Using a biopsy needle, the selected area was punched out, and a 3-mm tissue core was transferred to a 6 × 5 recipient block. Two tissue cores of invasive tumor were extracted to minimize extraction bias. Each tissue core was assigned a unique tissue microarray location number that was linked to a database containing other clinicopathologic data.

### 4.3. Immunohistochemistry

Detailed information of used antibodies in immunohistochemistry is listed in Table 5. All immunohistochemistry was performed with formalin-fixed, paraffin-embedded tissue sections using an automatic immunohistochemistry staining device (Benchmark XT, Ventana Medical System, Tucson, AZ, USA). Briefly, 5-µm-thick formaldehydefixed paraffin-embedded tissue sections were transferred onto adhesive slides and dried at 62 °C for 30 min. Standard heat epitope retrieval was performed for 30 min in ethylene diamine tetraacetic acid, pH 8.0, in the autostainer. The samples were then incubated with primary antibodies. After incubation with primary antibodies (amine oxidase related markers (LOX, AOC3, MAO-A, and MAO-B), molecular subtype related markers (ER, PR, HER-2, and Ki-67)), the sections were subsequently incubated with biotinylated anti-mouse immunoglobulins, peroxidase-labeled streptavidin (LSAB kit, DakoCytomation, Glostrup, Denmark), and 3,30-diaminobenzidine. Negative control samples were processed without the primary antibody. Positive control tissue was used as per the manufacturer’s recommendation and slides were counterstained with Harris hematoxylin.

### 4.4. Interpretation of Immunohistochemical Staining

All immunohistochemical markers were accessed by light microscopy. A cut-off value of 1% or more positively stained nuclei was used to define ER and PR positivity [43]. HER-2 staining was analyzed according to the American Society of Clinical Oncology (ASCO)/College of American Pathologists (CAP) guidelines using the following categories: 0 = no immunostaining; 1+ = weak incomplete membranous staining, less than 10% of tumor cells; 2+ = complete membranous staining, either uniform or weak in at least 10% of tumor cells; and 3+ = uniform intense membranous staining in at least 30% of tumor cells [44]. HER-2 immunostaining was considered positive when strong (3+) membranous staining was observed, whereas cases with 0 to 1+ were regarded as negative. Cases showing 2+ HER-2 expression were evaluated for HER-2 amplification by FISH. Ki-67 labeling index (LI) was defined as the percentage of total number of tumor cells with nuclear staining.

Immunohistochemical markers were accessed by light microscopy. The stained slides were reviewed and evaluated semi-quantitatively [45]. Tumor and stromal cell staining were assessed as 0, negative or weak immunostaining in <1% of the tumor/stroma; (1) focal expression in 1–10% of tumor/stroma; (2) positive in 11–50% of tumor/stroma; and (3) positive in 51–100% of tumor/stroma. This evaluation was performed over the entire tumor area and indicated as negative for 0 and positive for cases with a score > 1.

### 4.5. Tumor Phenotype Classification

In this study, we classified breast cancer phenotypes according to the immunohistochemistry results for ER, PR, HER-2, Ki-67 LI and fluorescent in situ hybridization (FISH) results for HER-2 as follows [46]: *Luminal A type*, ER or/and PR positive, HER-2 negative and Ki-67 LI < 14%; *Luminal B type*, (HER-2 negative) ER or/and PR positive, HER-2 negative and Ki-67 LI ≥ 14%; (HER-2 positive) ER or/and PR positive and HER-2 overexpressed or/and amplified; *HER-2 overexpression type*, ER and PR negative and HER-2 overexpressed or/and amplified; *TNBC type*: ER, PR, and HER-2 negative.

### 4.6. Functional Analysis Using STRING Database

We analyzed functional association of studied proteins using STRING database. As expression profile related genes are not available, we only were able to assessed functional enrichments using “Analysis” function of the platform. Adding other 10 first order proteins and second 10 order proteins did not change the clusters with fair local clustering coefficient of 0.799. As the available information of expression is limited in this study set, we investigated gene expression pattern of studied markers in 982 TCGA sequenced provisional samples via www.cbioportal.org by using “Mutual Exclusivity” function and the ‘Enrichments’ function. We performed pathway analysis using PANTHER database.

### 4.7. Statistical Analysis

Data were analyzed using SPSS for Windows, Version 23.0 (SPSS Inc., Chicago, IL, USA). For determination of statistical significance, Student’s *t* and Fisher’s exact tests were used for continuous and categorical variables, respectively. In the case of analyzing data with multiple comparisons, a corrected p-value with the application of the Bonferroni multiple comparison procedure was used. Statistical significance was set to *p* < 0.05. Kaplan-Meier survival curves and log-rank statistics were employed to evaluate time to tumor recurrence, disease-free survival (DFS) and overall survival (OS). Multivariate regression analysis was performed using the Cox proportional hazards model.

## Figures and Tables

**Table 1:** Clinicopathological characteristics of patients by breast cancer molecular subtypes.

Parameter	Total (*n* = 770) (%)	Luminal A (*n* = 380) (%)	Luminal B (*n* = 224) (%)	HER-2 (*n* = 68) (%)	TNBC (*n* = 98) (%)	*p*-Value
Age (years)						**0.040**
≤50	463 (60.1)	234 (61.6)	144 (64.3)	32 (47.1)	53 (54.1)	
>50	307 (39.9)	146 (38.4)	80 (35.7)	36 (52.9)	45 (45.9)	
Histological grade						**<0.001**
I/II	559 (72.6)	354 (93.2)	135 (60.3)	37 (54.4)	33 (33.7)	
III	211 (27.4)	26 (6.8)	89 (39.7)	31 (45.6)	65 (66.3)	
Tumor stage						0.158
T1	433 (56.2)	228 (60.0)	122 (54.5)	35 (51.5)	48 (49.0)	
T2/T3	337 (43.8)	152 (40.0)	102 (45.5)	33 (48.5)	50 (51.0)	
Nodal metastasis						**0.028**
Absent	460 (59.7)	213 (56.1)	133 (59.4)	43 (63.2)	71 (72.4)	
Present	310 (40.3)	167 (43.9)	91 (40.6)	25 (36.8)	27 (27.6)	
Estrogen-receptor status						**<0.001**
Negative	196 (25.5)	9 (2.4)	21 (9.4)	68 (100.0)	98 (100.0)	
Positive	574 (74.5)	371 (97.6)	203 (90.6)	0 (0.0)	0 (0.0)	
Progesterone-receptor status						**<0.001**
Negative	263 (34.2)	48 (12.6)	49 (21.9)	68 (100.0)	98 (100.0)	
Positive	507 (65.8)	332 (87.4)	175 (78.1)	0 (0.0)	0 (0.0)	
HER-2 status						**<0.001**
Negative	609 (79.1)	380 (100.0)	131 (58.5)	0 (0.0)	98 (100.0)	
Positive	161 (20.9)	0 (0.0)	93 (41.5)	68 (100.0)	0 (0.0)	
Ki-67 LI (%)						**<0.001**
≤14	457 (59.4)	380 (100.0)	49 (21.9)	17 (25.0)	11 (11.2)	
>14	313 (40.6)	0 (0.0)	175 (78.1)	51 (75.0)	87 (88.8)	
Duration of clinical follow-up (months, mean ± SD)	71.8 ± 21.7	72.9 ± 20.1	71.6 ± 21.6	64.1 ± 25.3	73.5 ± 24.4	0.019

TNBC: triple negative breast cancer; HER-2: Human growth factor receptor-2. LI: labeling index. Bold represents *p* < 0.05.

**Table 2:** Expression of the amine oxidase according to breast cancer subtypes.

Parameter	Total (*n* = 770) (100%)	Luminal A (*n* = 380) (49.4%)	Luminal B (*n* = 224) (29.1%)	HER-2 (*n* = 68) (8.8%)	TNBC (*n* = 98) (12.7%)	*p*-Value
LOX						0.178
Negative	413 (53.6)	218 (57.4)	113 (50.4)	31 (45.6)	51 (52.0)	
Positive	357 (46.4)	162 (42.6)	111 (49.6)	37 (54.4)	47 (48.0)	
AOC3						0.199
Negative	178 (23.1)	84 (22.1)	49 (21.9)	14 (20.6)	31 (31.6)	
Positive	592 (76.9)	296 (77.9)	175 (78.1)	54 (79.4)	67 (68.4)	
AOC3 (S)						<0.001
Negative	681 (88.4)	352 (92.6)	182 (81.3)	58 (85.3)	89 (90.8)	
Positive	89 (11.6)	28 (7.4)	42 (18.8)	10 (14.7)	9 (9.2)	
MAOA						<0.001
Negative	546 (70.6)	229 (60.3)	168 (75.0)	59 (86.8)	90 (91.8)	
Positive	224 (29.1)	151 (39.7)	56 (25.0)	9 (13.2)	8 (8.2)	
MAOB						0.020
Negative	658 (85.5)	337 (88.7)	189 (84.4)	57 (83.8)	75 (76.5)	
Positive	112 (14.5)	43 (11.3)	35 (15.6)	11 (16.2)	23 (23.5)	
MAOB (S)						0.075
Negative	648 (84.2)	331 (87.1)	178 (79.5)	55 (80.9)	84 (85.7)	
Positive	122 (15.8)	49 (12.9)	46 (20.5)	13 (19.1)	14 (14.3)	

S: stroma. Bold represents *p* < 0.05.

**Table 3:** Correlation of the expression of amine oxidase proteins breast cancer.

Parameters	LOX	AOC3	AOC3 (S)	MAOA	MAOB	MAOB (S)
LOX						
Correlation coefficient		0.238	0.144	0.001	0.060	0.160
*p*-value		<0.001	<0.001	0.982	0.098	<0.001
AOC3						
Correlation coefficient			0.160	0.087	−0.001	0.052
*p*-value			<0.001	0.016	0.978	0.147
AOC3 (S)						
Correlation coefficient				−0.035	−0.034	0.121
*p*-value				0.335	0.347	<0.001
MAOA						
Correlation coefficient					0.060	−0.043
*p*-value					0.095	0.233
MAOB						
Correlation coefficient						0.245
*p*-value						<0.001

**Table 4:** Univariate analysis of the impact of amine oxidase expression on breast cancer patient prognosis by log-rank test.

Parameter	Number of Patients/Recurrence/Death	Disease-Free Survival		Overall Survival	
		Mean Survival (95% CI) Months	*p*-Value	Mean Survival (95% CI) Months	*p*-Value
LOX			0.413		0.859
Negative	413/16/30	103 (102–105)		101 (99–103)	
Positive	357/10/25	105 (104–107)		102 (100–104)	
AOC3			0.062		0.050
Negative	178/10/19	103 (100–106)		100 (97–103)	
Positive	592/16/36	105 (104–106)		103 (101–104)	
AOC3 (S)			0.522		0.558
Negative	681/24/50	105 (103–106)		102 (101–103)	
Positive	89/2/5	106 (103–108)		104 (100–107)	
MAOA			0.484		0.074
Negative	546/20/45	104 (103–106)		101 (100–103)	
Positive	224/6/10	106 (104–107)		104 (102–106)	
MAOB			0.890		0.106
Negative	658/22/43	105 (104–106)		103 (101–104)	
Positive	112/4/12	105 (102–107)		100 (95–104)	
MAOB (S)			0.126		0.674
Negative	648/19/45	105 (104–106)		102 (101–104)	
Positive	122/7/10	103 (99–106)		102 (99–105)	

**Table 5:** Source, clone, and dilution of antibodies.

Antibody	Company	Clone	Dilution
*Amine oxidase*			
lysyl oxidase (LOX)	Abcam, Cambridge, UK	Polyclonal	1:100
amine oxidase (AOC3)	Abcam, Cambridge, UK	Polyclonal	1:1000
monoamine oxidase A	Abcam, Cambridge, UK	EPR7101	1:100
monoamine oxidase B	Abcam, Cambridge, UK	Polyclonal	1:100
*Molecular subtype-related proteins*			
ER	Thermo Scientific, San Diego, CA, USA	SP1	1:100
PR	DAKO, Glostrup, Denmark	PgR	1:50
HER-2	DAKO, Glostrup, Denmark	Polyclonal	1:1500
Ki-67	Abcam, Cambridge, UK	SP6	1:100

## Supplementary Materials

Supplementary materials can be found at www.mdpi.com/1422-0067/18/12/2775/s1.
